# Multi-omic analyses in immune cell development with lessons learned from T cell development

**DOI:** 10.3389/fcell.2023.1163529

**Published:** 2023-04-06

**Authors:** Martijn Cordes, Karin Pike-Overzet, Erik B. Van Den Akker, Frank J. T. Staal, Kirsten Canté-Barrett

**Affiliations:** ^1^ Department of Immunology, Leiden University Medical Center, Leiden, Netherlands; ^2^ Department of Biomedical Data Sciences, Leiden University Medical Center, Leiden, Netherlands; ^3^ Pattern Recognition and Bioinformatics, Delft University of Technology, Delft, Netherlands; ^4^ Novo Nordisk Foundation Center for Stem Cell Medicine (reNEW), Leiden University Medical Center, Leiden, Netherlands; ^5^ Department of Pediatrics, Leiden University Medical Center, Leiden, Netherlands

**Keywords:** multi‐omics, spectral flow cytometry, thymus, T cells, scRNA‐seq

## Abstract

Traditionally, flow cytometry has been the preferred method to characterize immune cells at the single-cell level. Flow cytometry is used in immunology mostly to measure the expression of identifying markers on the cell surface, but—with good antibodies—can also be used to assess the expression of intracellular proteins. The advent of single-cell RNA-sequencing has paved the road to study immune development at an unprecedented resolution. Single-cell RNA-sequencing studies have not only allowed us to efficiently chart the make-up of heterogeneous tissues, including their most rare cell populations, it also increasingly contributes to our understanding how different omics modalities interplay at a single cell resolution. Particularly for investigating the immune system, this means that these single-cell techniques can be integrated to combine and correlate RNA and protein data at the single-cell level. While RNA data usually reveals a large heterogeneity of a given population identified solely by a combination of surface protein markers, the integration of different omics modalities at a single cell resolution is expected to greatly contribute to our understanding of the immune system.

## 1 Introduction

Ever since the advent of early flow cytometry experiments in the 1980s, the field of immunology has been conducting research at the single cell resolution, intrinsically acknowledging the existence of heterogeneity present within populations of cells. Concomitantly, this field has been particularly welcoming for data-driven advances that form the basis of many novel discoveries in present day science.

The human immune system consists of many different cell types in different states depending on their stage of development, environmental stimulation, and interaction between non-immune cells (Tajer et al., 2019). Inside the cells, signal transduction cascades lead to activated transcription factors that initiate differentiation into a definite cell type by the activation of complex gene regulatory networks. Single-cell RNA sequencing of various tissues or cell populations has revealed the diversity and tremendously increased the resolution of cell types or states by capturing the transcriptomes of individual cells without the need of massive amounts of starting material. This has resulted in immunological studies resolving cellular heterogeneity (Villani et al., 2017; Zheng et al., 2017) and describing novel differences between differentiating cell populations. However, RNA expression does not always translate to the protein level and low RNA expression of transcription factors may give a distorted view of gene regulation within cells (Zhou et al., 2019).

In immunology, multi-color flow cytometry traditionally has been used to identify known and define new subpopulations of immune cells at the single cell level, based on combinatorial expression of mostly cell-surface markers at the protein level. In this way, flow cytometry constitutes a quantitative proteomics approach at the single cell level. At the molecular level, large amounts of multi-omics data in immunology and other fields of research have been generated in the form of bulk genomic, transcriptomic, proteomic an metabolomic datasets. While insightful, bulk data such as RNA-sequencing provides average expressions across a defined population therefore missing cell-specific developmental processes across lineage determining trajectories (Hu et al., 2018). To fully understand the human immune system, every aspect of the immune cells should be studied at multiple levels and time points. To detect all the differences between immune cells and to identify new populations it is important to link the gene expression to the protein (surface marker) expression of the cells.

In recent years multi-omic strategies have been developed for single-cell sequencing allowing to simultaneously profile the genome, transcriptome, and epigenome of the same cell (Villani et al., 2017; Zheng et al., 2017; Zhou et al., 2019). For T and B cells, T cell receptor and immunoglobulin information can also be obtained adding even more information to an already impressive amount of data that can be obtained from a single cell (De Simone et al., 2018). In this review we describe how scRNA-seq was used to identify rare thymus seeding cells within an already rare population of developing thymocytes (Cordes et al., 2022). We outline which considerations were taken designing the experiment, how to acquire the right cells from the right donors, which sequence protocol was used and how the analysis pointed us to the right populations. Importantly, we show that scRNA-seq data can guide multiparameter flow cytometry and cell sorting experiments leading to functional assays that validate results from scRNA-seq, which is a step that is not always taken in most of the current hypothesis-driven scRNA-seq studies.

## 2 Introduction to T cell development

**FIGURE 1:**
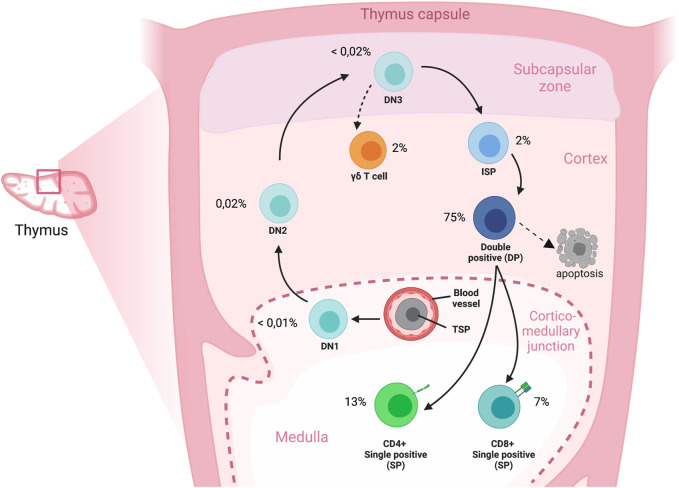
Overview of T cell development in the human thymus. Cross section of an adult thymic lobule representing the migration route of T-cell precursors during development. Immigrant precursors move to the thymus through blood vessels and enter near the corticomedullary junction; the TSPs subsequently migrate, and differentiate to DN, DP and finally to SP stages through the discrete microenvironments of the thymus. A directional reversal of migration back across the cortex towards the medulla occurs for the later stages of thymocyte development. The cell percentages represent the proportions of cell types compared to the total cell composition in the human thymus. DN: Double negative; DP: Double positive; ISP: Immature single positive; SP: Single positive; TSP: Thymic seeding progenitor.

The thymus is a primary lymphoid organ that provides a unique microenvironment for the development of T cells from hematopoietic stem or progenitor cells that originate from the bone marrow (Anderson and Jenkinson, 2001; Famili et al., 2017). Cells that seed the thymus go through different developmental stages that can be characterized by expression of different markers on the cell surface. The most immature thymocytes lack CD4 and CD8 expression (double-negative, DN). Following the DN stage, cells differentiate into CD4^+^CD8^+^ double-positive (DP) cells, after which they bifurcate to single-positive (SP) CD4^+^CD8^−^CD3^+^ or CD8^+^CD4^−^CD3^+^ stages that characterize mature T cells. Thymocytes proliferate and differentiate while they undergo T cell receptor (TCR) gene rearrangements and positive and negative selection, and these processes ultimately result in a diverse, yet tolerant TCR repertoire. In humans, the thymus is fully developed before birth in terms of the presence of all major thymocyte subpopulations, including the mature T cells. (see Figure 1) (Lobach and Haynes, 1987). To study thymocytes in an early state of their developmental trajectory it is important to select donors with sufficient DN cells. The rate of T cell production by the thymus is greatest during childhood, when the peripheral TCR repertoire is further established as a result of antigenic pressure. Several studies show that children until approximately 8 years of age have the highest percentage of DN cells (approximately 10% of total thymocytes) (Weerkamp et al., 2005). After puberty, thymocyte numbers drastically decrease, although the thymus remains functional for a lifetime (Bertho et al., 1997; Marusić et al., 1998; Jamieson et al., 1999; Poulin et al., 1999; Weerkamp et al., 2005).

Thymus microenvironmental signals including intercellular contact and cytokine signaling induce T cell development by activating specific transcriptional programs (Weerkamp et al., 2006b). The detection of these programs by measuring RNA expression in single cells enhances the understanding of T cell development when combined in a multi-parametric approach with surface marker protein expression. The sensitivity of detecting RNA expression in very rare cells is increased when rare populations are sorted or enriched prior to sequencing.

## 3 Considerations in study design

There are a number of important considerations to take into account when starting a multi-omics experiment. These include purification of cells under study, how to isolate such cells, what kind of sequencing protocol to use, how many cells to measure and how to analyze the wealth of data typically obtained from such experiments.

### 3.1 Enrichment of rare thymocyte populations

In general, purification of cells of interest allows for asking more defined questions. However, in doing so one cannot explore a complete map of all the cells that seem to play a role during development, and one may lose the proportionality of subpopulations. On the other hand, data from small subsets can be uncovered. There is another benefit, it might partially compensate for the differential cell death during data acquisition,—some cell types/states are more prone to die compared to others –, so by enriching these cells, some of their signal might still be recovered. In our example this is illustrated by a large difference in cell composition between the different developmental stages of T cell development. Within the thymus most of the thymocytes (around 80%) are DP cells, and only 10% of the cells are DN or SP cells (Weerkamp et al., 2005). So, if the biological effect of interest is limited to only a subpopulation of cells–such as DN cells–cell sorting is an important step to capture enough of the cells of interest. The most widely used high-throughput method used for enrichment of rare cell populations is flow-activated cell sorting (Radbruch and Recktenwald, 1995; Will and Steidl, 2010). Magnetic beads can be used for enrichment and manual isolation of specific tissue types or to remove unwanted cells such as dead cells, red blood cells, or non-immune cells such as epithelial cells and fibroblasts.

### 3.2 Cell caputuring for library generation

With droplet encapsulation-based methods using microfluidics, thousands of cells can be captured and barcoded in high-throughput. Single cells can be captured in a microwell or encased in water-in-oil droplets. Within the microwell or droplet barcoded cDNA is generated (Klein et al., 2015; Macosko et al., 2015). Commonly used solutions are Fluidigm C1, Biorad/Illumina ddSeq, Clontech ICell8, and BD Rhapsody for microwell encapsulation and inDrop, DropSeq, and 10X Chromium from 10X Genomics for droplet-based encapsulation. While microwell based methods are more versatile, precise and less labor-intensive, droplet systems like the 10X Chromium system are more used because they have enabled the capturing and barcoding of thousands of individual cells and thus have higher outputs (Gao et al., 2019). What must be taken into account is that 10X Single Cell Sequencing requires at least 500.000 cells in suspension to generate libraries. This can be a challenge when working with, for example, double-positive thymocytes of which most cells are apoptotic (Surh and Sprent, 1994).

### 3.3 Sequencing protocol

Multi-omic strategies are becoming popular in single-cell sequencing, as they increasingly allow for simultaneous analysis of multiple molecular modalities within individual cells. Omics assays that currently can be conducted at a single cell resolution include measurements on genomic, transcriptomic, or proteomic level. Increasingly, these methods can also be conducted on sequentially obtained tissue coupes, which thus yield information on the relative spatial positioning of the assayed cells. Collectively, these approaches have the promise to overcome limitations of integrating separate unimodal datasets and provides more comprehensive molecular information within individual cells (Ma et al., 2020). Particularly popular are multi-modal applications of single-cell RNA sequencing, which can be used to profile combinations of gene expression, splice isoforms, B- or T-cell receptor repertoire, as well as surface proteins.

Generally, there are two groups of single cell sequencing methods, plate-based and microfluidic-based techniques. Plate-based methods capture cells on multi-well plates or microfuge tubes. Microfluidic technology, on the other hand, captures cells in microfluidic droplets, is cost-effective, and allows for parallel quantification of gene expression profiles. Microfluidic methods can be continuous-flow (e.g., Fluidigm’s C1 system) or droplet-based (e.g., InDrop, Drop-seq, and 10X Genomics). Droplet-based platforms are easily automated and customizable for different experimental needs. Full-length scRNA-seq methods like SMART-seq2 and Cell-seq use well-based techniques but have limitations such as fewer cells per assay compared to droplet-based methods. Because every protocol can highlight different biological aspects of the cell, the choice of protocol should be carefully considered based on the research question. Full-length sequencing covers complete mRNA transcripts and increases the number of mappable reads enabling greater detection of low abundance transcripts, allelic gene expression analysis, and isoform discovery, (Ziegenhain et al., 2017), but requires more sequencing depth. In contrast, short-read methods create length reads of approximately 70–100 bases which can be sequenced from the 3′ or 5′ end of the transcript. Short-read based methods provide a powerful and cost-effective tool for the analysis of complex biological systems. Because of the high throughput, high accuracy and flexibility these protocols are the most widely used technology for genome and transcriptome sequencing. With both long and short read protocols unique molecular identifiers (UMI) are widely used because of the flexibility of multiplexing but also to reduce the effects of artifacts introduced by PCR amplification. The cDNA generation from both 3′ and 5′ based protocols begins with the capture of poly-A mRNA molecules and make use of a poly-dT primer for reverse transcription and a template-switching oligo (TSO) for reverse-transcription of 5′ transcripts. Of note, while capturing polyadenylated mRNA transcripts avoids capturing truncated mRNA transcripts, genomic DNA and rRNA it is subject to potential artifacts such as priming of intronic polyA-rich regions and incomplete cDNA production (Balázs et al., 2019).

To also capture information about the TCRs, the barcoded 5′ cDNA libraries can be used to enrich for the full V(D) J sequence by targeted PCR using reverse primers designed on the constant regions of alpha and beta chains (Matuła et al., 2020) or gamma and delta chains (Cordes et al., 2022). While 3′end sequencing approaches are standard practice for gene expression quantification, a recent comparison study between 3′ and 5′ 10X generated data shows that the 5′ assays captures more exonic UMIs and gene expression of genes with longer transcript lengths (Hsu et al., 2022). While V(D) J sequencing gives valuable information about the rearrangement processes of developing T cells, no actual protein data is investigated. To get more phenotypic insight, CITE-seq and REAP-seq offers an antibody-based phenotype analysis (Peterson et al., 2017; Stoeckius et al., 2017). With this method, antibodies against surface proteins of interest are coupled to a barcode, UMI and additional sequences that together are called antibody derived tags (ADTs). An additional library is then generated from these ADTs that can be sequenced independently but are often pooled with their respective cDNA library. With the development of the TotalSeq-C protocol (Biolegend) these three methods can all be combined in one go.

### 3.4 Cell numbers and sequencing depth

Two intertwined questions that should not be underestimated are the number of cells to sequence and the sequencing depth that is required per cell. Finding the right balance between these two factors is dependent on the scientific questions, but also on the sample availability and the experimental design. Generally, the number of cells to sequence is determined by the complexity of the sample (Svensson et al., 2017). In a sample with different amounts of cell types, 1 cell type dominates the population and rare cell types are only present in low fractions. The right experimental design can be a very important step to increase or decrease the heterogeneity of the sample. While enrichment of certain rare thymocyte subpopulations can increase heterogeneity between thymocytes (Dik et al., 2005), the removal of non-T cells (Weerkamp et al., 2006a) from the sample can remove unwanted heterogeneity and shift the focus to the detection of new rare thymic subpopulations (Cordes et al., 2022).

Comparisons between sensitivity and accuracy showed that full-length sequencing to detect transcripts with low gene expression requires 1 million reads per cell, while alternative splicing analysis may even require 30–60M reads per cell (Svensson et al., 2017; Ziegenhain et al., 2017). However, to perform unbiased cell-type identification within a mixed cell type population from 3′- or 5′-end libraries, a depth between 10.000 and 100.000 reads per cell can be sufficient (Pollen et al., 2014; Haque et al., 2017). For targeted approaches such as the V(D) J sequencing protocol of 10X Genomics, the recommended sequencing depth of 5000 reads per cell for 150bp reads will provide sequencing saturation of B- or T cell receptors.

### 3.5 Data analysis

During library construction, cDNA molecules from each cell are labeled with cellular barcodes and UMIs depending on the protocol used. A unique set of barcodes is used for every library and allow for the libraries to be pooled—or multiplexed—together in one sequence run. When a library is compiled of cells from different donor or conditions, Cell Hashing methods with barcoded antibodies can be used to add sample-specific sequence tags to correctly assign the cells after cell encapsulation, library preparation, and sequencing. Another option is *in silico* genotyping after sequencing by using single-nucleotide polymorphisms (SNPs) from the extracted data and using the overlapping SNPs between cells to determine the identity of the individual donors (Huang et al., 2019). For optimal results, aforementioned approaches are often combined.

#### 3.5.1 Quality control and normalization

Forthcoming reads obtained in single-cell sequencing experiments need to be assigned to their cells through a process known as “demultiplexing”. In this process, reads are grouped based on their cellular barcode followed by alignment to a reference genome. This results in a count matrix with a dimension of number of barcodes multiplied by the number of genes. Before further analyzing the gene expression data, quality control of the data has to be performed to be sure that only biologically relevant cells are used for downstream processing (Luecken and Theis, 2019). Most filtering strategies are based on three data covariates, 1—the count depth per cell, which is calculated by counting the number of transcripts with the same barcode; 2—the number of genes expressed per cell; 3—the fraction of mitochondrial genes expressed per cell (Ilicic et al., 2016; Griffiths et al., 2018). Cells that only show expression of a few genes and a high fraction of mitochondrial gene expression are most likely apoptotic cells in which only the mitochondrial mRNA is conserved after the cytoplasmic mRNA has leaked through the membrane (Galluzzi et al., 2012). On the other hand, cells with an unusually high-count depth per cell combined with an unusual high number of genes expressed per cell can be an indication that these are doublets, meaning that there have been 2 cells with the same barcode captured in one oil droplet.

When cells from different donors were pooled prior to sequencing, *in silico* generated genotype information can also be used to identify if barcodes are shared across genotypes (Huang et al., 2019).

The three mentioned quality covariates can be visualized to determine which cells are to be removed. By plotting the metric-distributions outliers can be identified by using appropriate thresholds. These thresholds are user-defined and should be carefully considered to prevent misinterpretation which can lead to removing biologically relevant data by non-experienced users. Choosing the right parameters for filtering should be first and foremost be driven by the investigated biological system set for every individual dataset before combining into final analysis. The importance of using different quality control (QC) thresholds was described in a recent study by Subramanian et al. which showed that QC metrics vary by cell type within a tissue and using fixed QC threshold for mitochondrial genes can lead to loss of cell subsets within a dataset (Subramanian et al., 2022). Another option is to visualize by generating a Uniform Manifold Approximation and Projection (UMAP) or *t*-distributed Stochastic Neighbor Embedding (*t*-SNE) before filtering and perform clustering analysis to investigate clusters; as in most cases, poor-quality cells can be removed because they cluster separately from good-quality cells (Stegle et al., 2015). Several technical factors such as gene length, GC-content, sequencing depth, and dropouts can introduce bias into raw read counts during sequencing, leading to inaccurate representation of biological gene expression. This noise from technical differences between sequencing platforms could obscure underlying biological differences between samples, making interpretation from unnormalized data unreliable. While UMI-based protocols minimize amplification biases, normalization at cell and gene levels is necessary to correct for technical biases and sampling effects. Log normalization is commonly used in UMI-based data analysis (Stuart et al., 2019), while non-UMI data employs normalization methods such as CPM, RPKM, TPM, and FPKM (Vallejos et al., 2017). However, advanced normalization strategies are required due to scRNA-seq data’s intricacies, such as data sparsity and high heterogeneity.

#### 3.5.2 Integration and regression

Integrating data and regression are two different methods of controlling for biological or technical covariates in single-cell RNA sequencing data to remove unwanted variation that arises from combining different datasets, protocols, and sequencing methods. Correcting for such variation helps reveal important biological signals and processes. However, data correction for biological effects may not always be in the best interest, and correction for one effect may mask another. Thus, it is advised first to evaluate the study’s objective and context before deciding on data correction measures (Luecken and Theis, 2019).

Data integration is a method of combining multiple datasets into a single integrated dataset, so that the unwanted variation is effectively removed from each dataset. The idea behind this method is to correct for any technical or biological factors that might impact gene expression, so that the true gene expression signals can be analyzed. With the rise of scRNAseq datasets methods for integration also have been developed widely as described in this benchmarking study by (Luecken et al., 2022). Regression of biological signals, on the other hand, is a method of removing the influence of a specific set of biological variables on the expression of a set of genes. This is typically done using regression models, such as linear regression against a cell cycle score, which is a way to remove the effects of the cell cycle. By regression the relationship between the gene expression data and the biological variables is estimated. The idea behind this method is to control for the effects of the biological variables so that they do not impact the analysis of the gene expression data.

In some cases, highly expressed genes encoding for specific biological processes may bias the results of an unsupervised clustering. For example, a study by Sundell et al. found that including B−/T-cell receptor genes resulted in clusters primarily defined by variations in *V*-gene segment expression. Excluding these genes before clustering led to biologically more meaningful subsets such as memory B and memory T cells. (Sundell et al., 2022). Similarly, genes related to metabolic changes like mitochondrial, ribosomal and histone genes might obscure the detection of biologically relevant subsets (Subramanian et al., 2022). In general, the choice between integrating data to remove biological effects or using regression to remove biological signals depends on the specific goals of the analysis and the nature of the data. In most cases multiple variations of the above should be tested to determine the right analysis protocol for each individual dataset.

#### 3.5.3 Dimensionality reduction

The scRNA-seq data, after filtering out poor samples, can be interpreted through a vast array of bioinformatic and computational techniques, as extensively reviewed in Poirion et al. (2016). Despite the high-dimensional nature of scRNA-seq expression metrics, not all data dimensions are necessary for meaningful classification of cellular expression profiles, which can be effectively explained through a reduced number of dimensions focusing on relevant biological signals. Many methods aim to simplify these multi-dimensional data, represented by each dimension being the expression of a single gene, into a smaller number of dimensions for easier visualization and interpretation. Dimensionality reduction (DR) techniques, such as Principal Component Analysis (PCA) (Wold et al., 1987), t-distributed Stochastic Neighbor Embedding (*t*-SNE) (Van der Maaten and Hinton, 2008), Uniform Manifold Approximation and Projection (UMAP) (McInnes et al., 2018), Self-Organizing Maps (SOM) (Kohonen, 2012), and Model embedded dimension reduction, allow for improved data visualization and resolve the issue of data sparsity. An effective low-dimensional representation should summarize the data in a few optimal dimensions while preserving the underlying structure of the dataset and describing its variability. These DR techniques, particularly PCA, have proven to be valuable tools for examining heterogeneity in scRNA-seq data, with further advancements incorporating various machine learning algorithms (Petegrosso et al., 2020). PCA on its own is not optimal for data visualization as it does not take into account the sparsity of the data and can lead to a misleading representation of the data structure. *t*-SNE, on the other hand, is better suited for this purpose due to its graph-based non-linear approach and is often used in conjunction with PCA. It uses a probabilistic distance model to establish relationships between high dimensional data points, which are then optimized and reconstructed in lower dimensions using gradient descent. While *t*-SNE is effective for visualizing non-linear datasets and preserving local structures, it may overlook the global structure and potentially lead to misinterpretation of differences between cell populations. To address these limitations, the UMAP technique has emerged as a more favorable DR method. UMAP is similar to t-SNE but also preserves the global structure, making it a more comprehensive tool for investigation lineage differentiation.

In a highly heterogeneous dataset, it is important to carefully consider the variance added to generate a *t*-SNE or UMAP. While generating an elbow plot or using the permutation-test-based jackstraw method (Chung and Storey, 2015; Macosko et al., 2015) maybe helpful in identifying the statistical significance of individual principal components (PCs), this will not automatically determine which PCs provide relevant variance. Including more PCs will highlight more of the biological signal while the same signal can noise the true signal. Lowering the number of PCs will shift the focus to the major variance factors while interesting, underrepresented factors that are pushed into lower PCs will be discarded from further downstream analysis. In the end, the scientific question should be leading, also in data visualization.

#### 3.5.4 Where biology gets in the way

In our recently published scRNAseq dataset (Cordes et al., 2022) from six individual healthy thymus samples, integration, regression, and gene removal were used to correct for biological process not fitting into the research scope which was learning more about the differentiation of early thymocytes.

The thymus is an example of a biological system containing a very heterogeneous population of differentiating cells, requiring different analytical approaches then the standard protocols. In the thymus >90% of the cells die during the different selection steps of thymocyte development (Surh and Sprent, 1994). One would expect more mitochondrial expression in such a dataset. Generally speaking, cells are removed from the dataset when the proportion of mitochondrial gene expression is above 5%–10% within a cell (Brennecke et al., 2013). Two studies on similar cell types, thymic progenitor populations (Lavaert et al., 2020) and our own study (Cordes et al., 2022), using the same techniques showed different results. Lavaert et al., identified two potential thymic seeding progenitor populations within a scRNA-seq dataset where cells >10% mitochondrial expression were removed, while we identified a third progenitor population using a higher threshold (>30%) which has the ability to develop into T cells much faster which would have been missed when the threshold was set to 10%. The cells with a higher mitochondrial gene expression also expressed anti-apoptotic and proliferation genes, indicating that they were not dead.

For the integration of our different samples the method Canonical Correlation Analysis (CCA) (Butler et al., 2018) was applied, which successfully removed individual-specific clustering. Because the samples were a mix of male and female donors, sex related interference was still found in our data with genes like the *X*-linked gene XIST within the top differentially expressed genes between clusters. Removal of XIST from the count matrix also overcame this gender-related issue. Mitochondrial, ribosomal and histone genes were also removed to prevent clustering based on metabolic activity of the cells. While cell cycle regression prevented clusters of cells in the same proliferation phase, a clear separation could be seen between cycling cells and cells with high activity of processes related to the rearrangement of TCR genes. This shows that major biological events within a biological system, like proliferation and cell death during T cell development will be detectable within such a dataset regardless of correction (Lavaert et al., 2020; Le et al., 2020; Park et al., 2020; Cordes et al., 2022).

Also when performing dimensionality reduction different settings highlighted different biological processes of the human thymus; the UMAP from Figure 1 in Cordes et al., 2022 displays the overall lineage differentiation of T-cells in a UMAP generated using 10 PCs while the same dataset was used also for cell type identification using a UMAP generated by > 35 PCs in Figures 3 and S4 in Cordes et al., 2022.

## 3.6 Integration with existing databases to verify and annotate subsets

**FIGURE 2:**
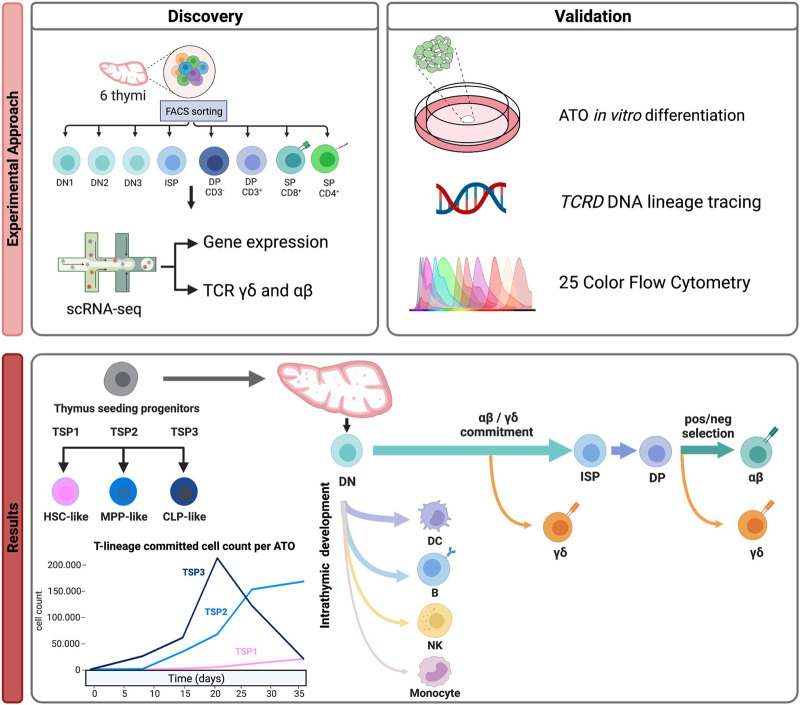
An example of a multi-omics approach to understand human T cell development (Cordes et al., 2022). Discovery: Purification of thymocyte subsets isolated from 6 healthy donors to generate a multi-omic (gene expression and γδ/αβ TCR) scRNAseq dataset resulting in the identification of different thymus seeding progenitor populations and detection of multiple lineage differentiation trajectories (including alternative lineages). Validation: by functional assays, lineage tracing and multi-color flow cytometry.

Immune development intrinsically takes place in several organs. Hence, to be able to appreciate the full developmental trajectory of particular immune subsets, from–from cradle to *in situ* effector -, we generally need to integrate single-cell studies created from different immune tissues. This helps contextualizing cell populations found in one tissue and links it to populations in other tissues. While the gene expression profiles of each population are often indicative, integration with existing annotated data can also provide important insight, not only in the nature of the subsets but also in their developmental or cation status. In our study on T cell development, integration with existing data helped infer developmental pathways and illustrate alternative, non-T cell lineage pathways (see Figure 2).

## 3.7 Functional genomics

The combination of many flow cytometry parameters in one sample allows to visualize many (sub) populations and may reveal rare populations that are missed in conventional flow cytometry using a limited number of parameters. While conventional flow cytometry uses one detector to measure the emission maximum of each fluorophore, spectral cytometry uses all the detectors that are available to measure the entire emission spectrum. This allows the use of many more markers compared to conventional flow cytometry because the un-mixing of spectral signatures helps resolve overlapping fluorescence spectra. However, in both approaches the anatomical and spatial interactions of cells are lost because the tissue is processed into a single cell suspension. New techniques using spatial proteomics [for example, mass cytometry imaging in tissue slices, or tissue cytometry by time of flight (tissue CyTOF)] would supplement flow cytometry data with the spatial localization of certain cell populations in a tissue, especially when combined with spatial transcriptomics, ideally integrated with scRNA-seq (Longo et al., 2021).

We have outlined how cell enrichment (for example, by flow-activated cell sorting) of rare populations can guide the interpretation of and even reveal scRNA-seq data leading to conclusions that otherwise would have been missed. Just as important is the follow-up by testing and validating the new-found data in wet-lab experiments. For example, in our dataset (Cordes et al., 2022) we found RNA expression of several candidate surface markers that—in combination with other existing markers—would ease the sorting of the newly identified rare populations. In practice, however, RNA and protein expression do not always correlate (Li et al., 2020). Thus, each candidate needs to be tested for protein expression. Also, cell enrichment using the new-found candidate markers to sort the new rare populations and subsequent functional validation in an *in vitro* assay is required to validate that the new markers have contributed to the isolation of the novel rare (sub) populations. Therefore, single-cell RNA-seq data can guide multiparameter flow cytometry and cell sorting experiments and should lead to functional assays that validate the *in silico* results.

## 4 Conclusion

**FIGURE 3:**
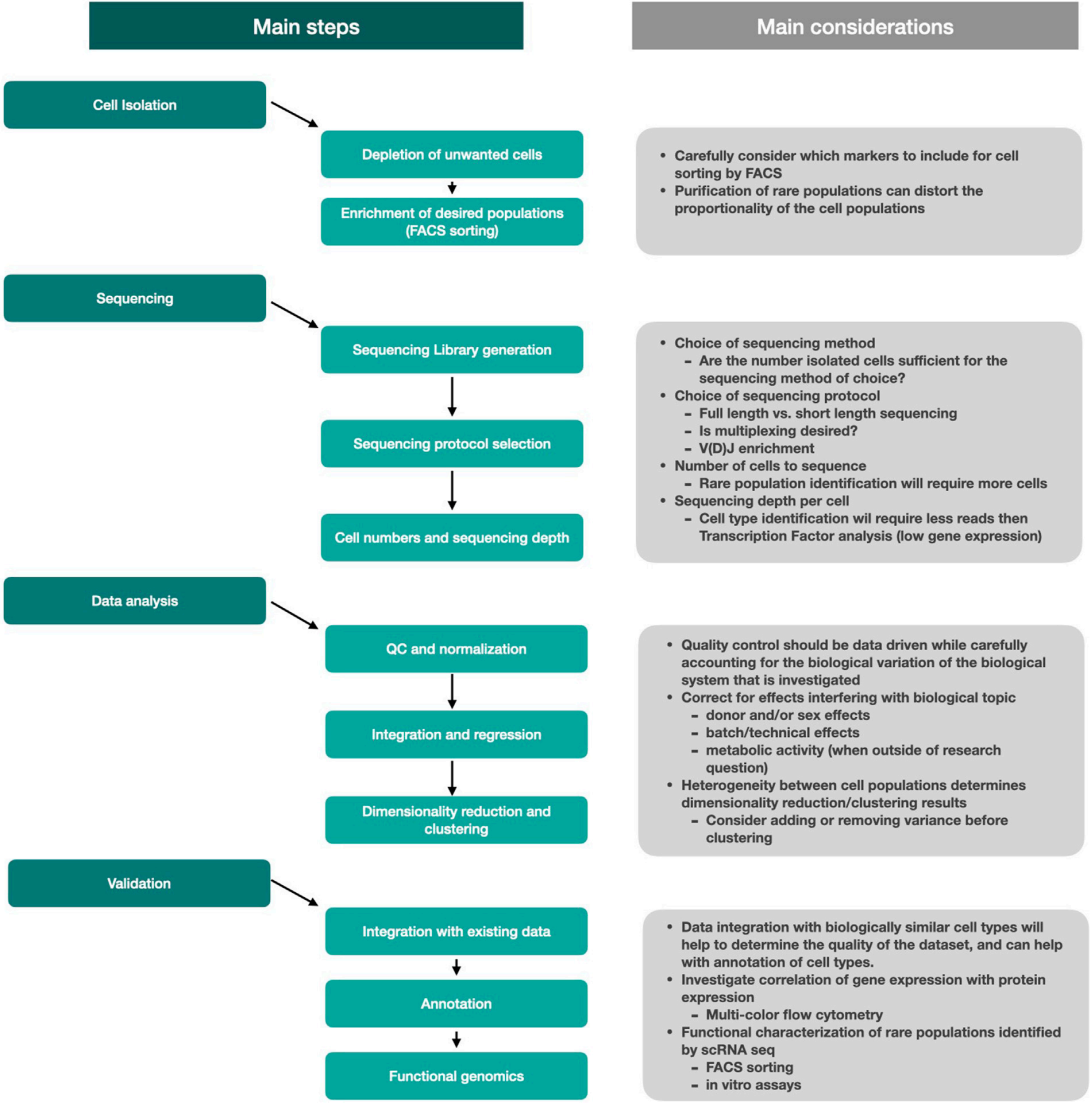
Schematic overview of the main steps and considerations used to perform studies on rare cells.

In conclusion, the detection and analysis of rare subsets of cells requires several critical steps to be considered (Figure 3). Rare cell isolation is most efficient when unwanted cells such as dead cells, red blood cells, but also unwanted cells that are abundantly present in the sample, are removed upfront. Further enrichment of rare cells is most commonly achieved using fluorescence-activated cell sorting. The choice of sequencing method should be considered based on the biological question, but also depends on the number of cells that can be isolated from your tissue. Sequencing itself will generate the final dataset and while a broad range of analysis tools are available to help and investigate the data, the choice of sequencing protocol will be vital for the end result. Data analysis will help uncover new biological insights, but one should carefully consider the quality control and analysis steps to ensure that the biology of interest will not be masked by non-relevant biological effects. Integration of new populations with existing datasets will help with cell type identification while functional assays can tell more about phenotypic properties and biological function of cells.

Single-cell RNA seq has enabled the identification of rare cells with distinct gene expression, and thus increased our understanding of the complex heterogeneity of cell types and their interaction in what previously seemed homogeneous cell populations. Integration of data from various sources and databases can lead to new discoveries or provide evidence for connections between cells. For instance, the integration of CD34^+^ BM data with similar data from the thymus helped in the identification of the three types of cells that seed the human thymus (Cordes et al., 2022). The strength of RNA-Seq is immensely enhanced when integrated with other techniques at the single cell level, including those that mark protein expression on the cell’s surface, such as flow cytometric approaches. This not only provides independent validation of results by another omics platform, but also can provide leads for further functional studies. Therefore, the integration of omics data from different immune subsets combined with integration of various high data content platforms (e.g., scRNA-seq, ATAC-seq, multi parameter flow cytometry or mass cytometry) allow an unprecedented deep immune profiling that leads to new biological insights.
